# A Sensor for Characterisation of Liquid Materials with High Permittivity and High Dielectric Loss

**DOI:** 10.3390/s22051764

**Published:** 2022-02-24

**Authors:** Chen Wang, Xiaoming Liu, Zhixiang Huang, Shuo Yu, Xiaofan Yang, Xiaobang Shang

**Affiliations:** 1 School of Physics and Electronic Information, Anhui Normal University, Wuhu 241002, China; wangchen@ahnu.edu.cn (C.W.); yushuo95@ahnu.edu.cn (S.Y.); 2 School of Electronic and Information, Anhui University, Hefei 230601, China; zxhuang@ahu.edu.cn; 3 The State Key Laboratory of Complex Electromagnetic Environment Effects on Electronic and Information System, Luoyang 471004, China; xiaofan_uestc@sina.com; 4 The National Physical Laboratory, Teddington TW11 0LW, UK; xiaobang.shang@npl.co.uk

**Keywords:** dielectric sensor, high permittivity, high loss, liquid, complementary split ring resonator

## Abstract

This paper reports on a sensor based on multi-element complementary split-ring resonator for the measurement of liquid materials. The resonator consists of three split rings for improved measurement sensitivity. A hole is fabricated at the centre of the rings to accommodate a hollow glass tube, through which the liquid sample can be injected. Electromagnetic simulations demonstrate that both the resonant frequency and quality factor of the sensor vary considerably with the dielectric constant and loss tangent of the liquid sample. The volume ratio between the liquid sample and glass tube is 0.36, yielding great sensitivity in the measured results for high loss liquids. Compared to the design based on rectangular split rings, the proposed ring structure offers 37% larger frequency shifts and 9.1% greater resonant dips. The relationship between dielectric constant, loss tangent, measured quality factor and resonant frequency is derived. Experimental verification is conducted using ethanol solution with different concentrations. The measurement accuracy is calculated to be within 2.8%, and this validates the proposed approach.

## 1. Introduction

Complex permittivity is one fundamental electromagnetic property of dielectric materials [1]. Accurate dielectric measurements play an important role in applications such as manufacturing [2], processing [3], antenna design [4] and aerospace technology [5], etc. Usually, the permittivity needs to be known as a priori in, for example, the design of microwave planar circuits. Compared to solid materials, the permittivity of liquid materials is prone to impacts from factors such as temperature, moisture, contaminants in the measurement sample holder, air pressure, and so on [6,7]. In addition, the measurement of a liquid sample is less convenient due to its fluid nature. Many liquid samples usually consist of polar molecules, leading to high dielectric constant and loss of properties. In this regard, in situ measurement of dielectric constant and loss tangent for liquid materials is in active demand.

Complex permittivity may be expressed as $\varepsilon_{r}=\varepsilon_{r}^{\prime }-j\varepsilon_{r}^{\prime \prime }=\varepsilon_{r}^{\prime }\left(1-j\tan\delta \right)$, where $\varepsilon_{r}^{\prime }$ is the real part and often referred to as dielectric constant, $\varepsilon_{r}^{\prime \prime }$ is the imaginary part and is called the loss factor, while tanδ is the loss tangent characterising the dielectric loss. The precise measurement of complex permittivity requires the determination of both the dielectric constant and loss tangent with sufficiently high accuracy.

There exist various types of measurement methods for liquid samples, including capacitance method, transmission line method, free-space method, and resonant cavity method. A cross capacitance method was developed for the accurate measurement of liquids of low dielectric constants in Ref. [8]. In general, capacitance methods are suitable for low frequency measurements. The transmission line method requires that the material be placed in a part of the enclosed transmission line during measurements [9,10]. It also requires precisely manufactured samples to fit the transmission line [11]. In addition, the injected liquid sample in an enclosed transmission line always faces the challenge of sealing the sample without leakage. The free space method uses free space as the transmission medium and is more suitable for dielectric measurement in the millimeter wave range [12]. The resonant cavity method is sensitive to low-loss and low-permittivity samples with high accuracy. The traditional resonant method usually creates a higher mode for high permittivity samples [13]. Moreover, high-loss samples usually degrade the quality factor, resulting in a considerable reduction of measurement accuracy [14]. In summary, new dielectric measurement techniques shall be developed for high-permittivity and high-loss liquid samples with sufficiently high sensitivity.

In recent years, microwave sensors using the resonance method have become increasingly popular. Structures such as the split-ring resonator (SRR), complementary spiral resonator (CSR), and complementary split ring resonator (CSRR) are the most commonly used ones in the design of microwave liquid sensors [15,16,17,18,19,20,21,22,23,24,25,26,27,28,29,30]. Examples of SRR structure can be found in Refs. [15,16,17,18,19,20]. The sensor in Ref. [15] must maintain the fluidity of the liquid during measurement, which may cause a lot of loss of liquid samples. Sensors reported by Kiani et al. [16] can measure the dielectric constant of liquids, but not the loss tangent. This work was later further developed by the same group to realize dual-frequency operation [18]. Juan et al. [17] developed a concentration sensor using the Q factor and the maximum *S*_21_ of the resonance as sensing magnitudes. Juan et al. [19] modified this design using certified biocompatible material. The structure was fabricated by 3D printing, which provides stronger interaction between the electromagnetic fields and the sample. The microfluid sensor reported in Ref. [20] can measure both the real and imaginary parts by using both magnitude and phase of *S*_21_. However, the accuracy is not good enough, which may be attributed to the fact that that phase is more prone to measurement errors. Interestingly, Su et al. [21] made a systematic study on defect-ground-plane based resonators. This general method provides guidelines for the design of microstrip line resonators. Refs. [22,23] reported on sensors based on CSR structures. The sensor reported by Su et al. [22] uses flexible materials but can only measure low-loss materials, while, the sensitivity of the sensor in Ref. [23] is too low and much noise can be observed during in vivo measurement. The CSRR structure can be found in Refs. [24,25,26,27,28,29,30]. The substrate integrated waveguide structure reported by Cai et al. [24] can only measure the real part of the permittivity. The sensor developed by Chen et al. [25] measured permittivity through the influence of liquid on the operating frequency of an antenna. However, the measurement was greatly influenced by its environment and the deviation is as high as 11.0%. Good sensitivity can be obtained using the sensors in Refs. [26,27,28], but the measurement range of the loss tangent is narrow, and they cannot measure corrosive liquids for direct contact between the sensors and samples. In Ref. [29], liquid samples pass through the central hole of the CSRR sensor, which maximizes the sample’s exposure to the electric field. However, it is still difficult to distinguish small changes in permittivity. Another CSRR-based liquid sensor working at 200–330 MHz is reported for dielectric measurement of different density ethanol [30]. Again, it is not able to measure corrosive liquids.

For the resonance-based method, the resonant structure acts as an LC resonator. The general description of this type of sensor can be found in Ref. [21] and a good review on this topic can be found by Juan et al. [31]. When materials are placed on top of the resonator, interaction between fields and samples will modify the resonant frequency $f_{r}$ and the corresponding quality factor *Q*. The permittivity can be retrieved from the variation in $f_{r}$ and *Q*. However, the difficulty in the measurement of high-permittivity high-loss measurement lies in its dilemma with high sensitivity. Being inspired by two pioneered designs in Ref. [29] and Ref. [32], we recognize that two approaches may be beneficial to handle this dilemma. One is to increase the field intensity in the resonating area under the condition of unit power input. The other is to use partial filled sample. By combining these methods, the sensitivity and measurement range can be simultaneously improved.

**Figure 1:**
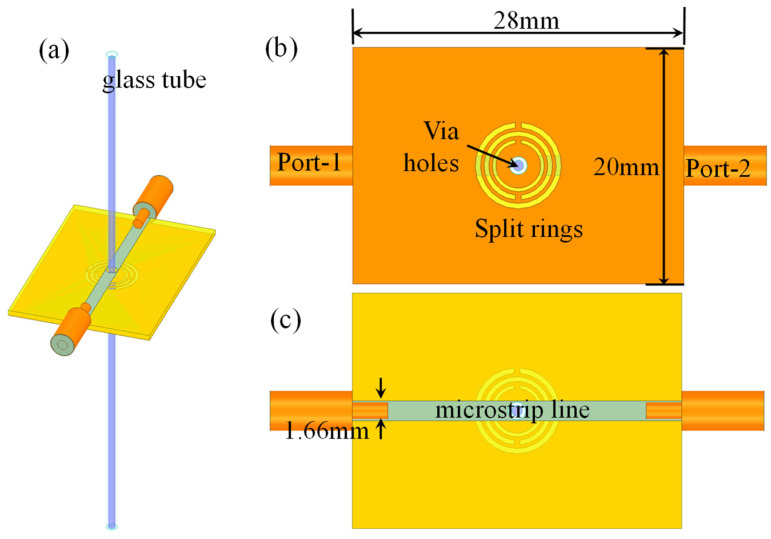
Circular CSRR (**a**) a 3D illustration; (**b**) Top view; (**c**) Rear view.

To enable measurement of high-loss polar liquids, a multi-element CSRR structure is proposed. In this design, three circular split rings are used, which will prove that ahigher regional electric field can be created. A glass tube passes through the central hole of the coaxial rings, see Figure 1. The glass tube is made of low loss fused quartz, functioning as a liquids holder and partial filling the resonator. These methods enable high sensitivity measurement for high-permittivity and high-loss liquids.

In the following sections, the performance of the circular CSRR structure and the rectangular CSRR structure will be compared in detail in Section 2. It is concluded that the circular CSRR structure has higher sensitivity than the rectangular CSRR structure. In Section 3 and Section 4, a theoretical model is established based on the simulation results, and extensive simulation analysis is carried out. Measurements and results are presented in Section 5. Section 6 summarizes this work.

## 2. Resonant Properties of CSRR Structures

This section provides some physical insights into CSRR. A comparison between circular and rectangular CSRR is also presented.

**Figure 2:**
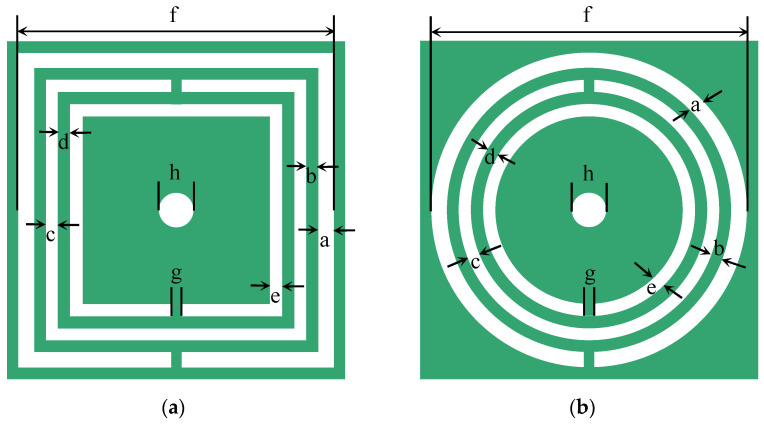
CSRR based on (**a**) rectangular structures; and (**b**) circular structures.

The CSRR structure in Ref. [29] is shown in Figure 2a, where the resonator uses three rectangular split rings. In the middle of the CSRR, a via hole is drilled to cater a glass tube. The main parameters of this structure are given in Table 1. The initial values of these parameters are chosen so that the outer ring is half wavelength at the resonant frequency. The final values are obtained through parameter sweep in HFSS software. Such a structure can be modelled as a hybrid LC resonator [33,34]. The corresponding values of each lumped element can be retrieved from full wave simulations, and the extracted values are given in Table 2. The response of the LC resonant circuit is evaluated using both circuit method and full wave simulation. It has to be mentioned that the central frequency is largely determined by the length of the split ring. Changing the length will modify the central frequency. To make a comparison to the design in Ref. [29], we chose the diameter of the circular ring to be the same as the side length of the rectangular ring.

According to the equivalent circuit diagram, the resonant frequency of the structure can be deduced as [35]

(1) $$f_{r}=\frac{1}{2\pi \sqrt{L_{r}(C_{c}+C_{r})}}$$

**Figure 3:**
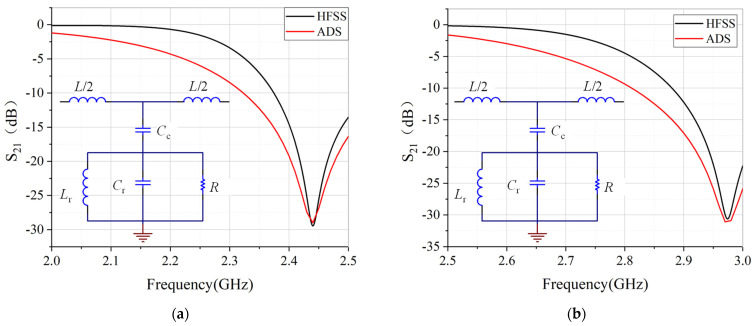
The simulated transmission results of (**a**) Rectangular rings; and (**b**) Circular rings. The insets are their corresponding equivalent circuit models.

**Figure 4:**
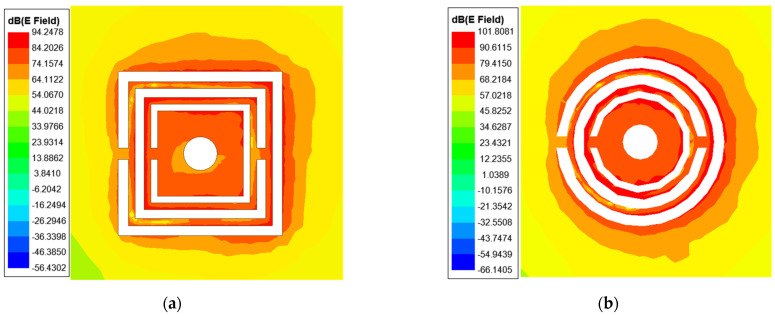
The simulated field distribution of (**a**) Rectangular rings; and (**b**) Circular rings.

The equivalent circuit model gives good prediction to the resonant frequencies, compared to the full wave simulated results by commercial software HFSS, as shown in Figure 3. The resonant dips of the circular CSRR are several dB larger than that of the rectangular CSRR. The field distribution around the resonator is plotted in Figure 4. It can be seen from the scale bar that the peak field of the circular CSRR is 7 dB higher than that of the rectangular CSRR, indicating that better interaction between fields and sample can take place for circular CSRR. This may explain why circular CSRR has deeper resonance.

## 3. Sensitivity Analysis

The sensor structure and some dimensions are shown in Figure 1. The substrate is 20 mm wide and 28 mm long. Port 1 and Port 2 are connected to coaxial lines. The quartz glass tube has a length of 75 mm, an outer diameter of 1.5 mm, and an inner diameter of 0.9 mm. The volume ratio between liquid sample and glass tube is 0.36. Simulations of liquid samples of different concentrations are undertaken. During the measurement, the samples are injected into the glass tube using a syringe. Each glass tube is used for only one concentration to avoid contamination.

### 3.1. Resonant Frequency

**Figure 5:**
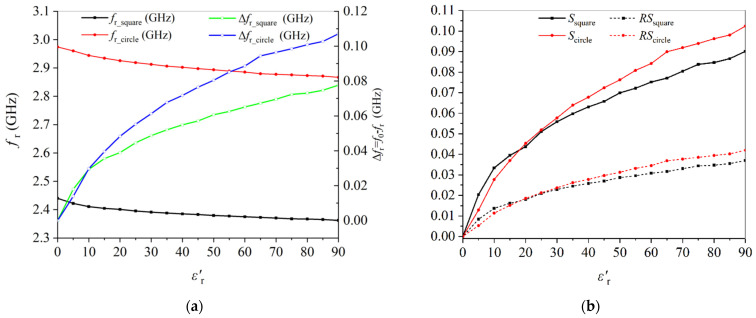
Comparison of sensitivity of the two resonators. (**a**) frequency and frequency shift, (**b**) sensitivity versus change in dielectric constant.

To compare the sensitivities of the two structures, the resonant frequencies ($f_{r}$) and frequency shifts ($\Delta f_{r}=f_{r}-f_{0}$, with $f_{0}$ defined as the resonant frequency at unloaded case) versus dielectric constant ($\varepsilon_{r}^{\prime }$) are plotted in Figure 5. Firstly, the unloaded sensor with an empty glass tube is simulated. Then the glass tube filled with samples of different $\varepsilon_{r}^{\prime }$ in the range of 1–90 is simulated. The resonant frequencies and the frequency shift relative to the unloaded case are recorded.

As seen in Figure 5a, the resonant frequencies for the circular and rectangular CSRR structure are 2960 MHz and 2440 MHz, respectively, which is consistent with the results of the equivalent circuit in Figure 2. The frequency shift when $\varepsilon_{r}^{\prime }=90$ of the circular CSRR structure is 110 MHz, and it is 80 MHz for the rectangular CSRR structure. The frequency shift is increased by 37%. According to the definition by Juan et al. [31], the relative sensitivity (*RS*) in this work can be written as

(2) $$RS=\frac{\Delta f_{r}}{f_{0}\cdot (\varepsilon_{r}^{\prime }-1)}\times 100$$

where $f_{0}$ is the resonant frequency at unloaded case, $\Delta f_{r}$ is the frequency shift, and $\varepsilon_{r}^{\prime }$ is the dielectric constant. Sensitivity (*S*) may be defined by $S=\left(\Delta f_{r}/f_{0}\right)\times 100$, but the relative sensitivity seems better since *RS* involves the quantity to be measured. The calculated *RS* of circular structure is 0.041, and it is 0.036 for the rectangular structure, as shown in Figure 5b. It is seen that the sensitivity is increased by 13.9%. Therefore, it can be deduced that the sensitivity of the circular CSRR structure is better than that of the rectangular one. All other parameters being equivalent, higher *RS* means higher sensitivity. Actually, the increase in sensitivity may be attributed to the increase in electric intensity, as shown in Figure 4. The circular sensor concentrates more power in the central hole, where the liquid sample is placed, so that the field interaction with sample can be observed. Therefore, for measuring liquid samples with $\varepsilon_{r}^{\prime }$ in the range of 1–90, the circular CSRR structure is a better choice, particularly in the high-permittivity range.

### 3.2. Quality Factor

In many cases, the permittivity is expressed using dielectric constant and imaginary part. The imaginary part of the complex permittivity of the material under test (MUT) can be expressed by loss tangent, which can be written as [19]:

(3) $$Q_{\mathrm{MUT}}=\frac{1}{\tan\delta }=\frac{\varepsilon_{r}^{\prime }}{\varepsilon_{r}^{\prime \prime }}$$

where, $Q_{\mathrm{MUT}}$ can be calculated by unloaded quality factor $Q_{U}$ and the corresponding $S_{21}$ [36]

(4) $$Q_{\mathrm{MUT}}=Q_{U}\left[1-10^{\frac{S_{21}(\mathrm{dB})}{20}}\right]$$

Therefore, the loss tangent can be calculated by using the quality factor *Q* and the transmission coefficient $S_{21}$. It is deduced that the larger variation in $S_{21}$ ($\Delta S_{21}$), the higher the measurement sensitivity.

**Figure 6:**
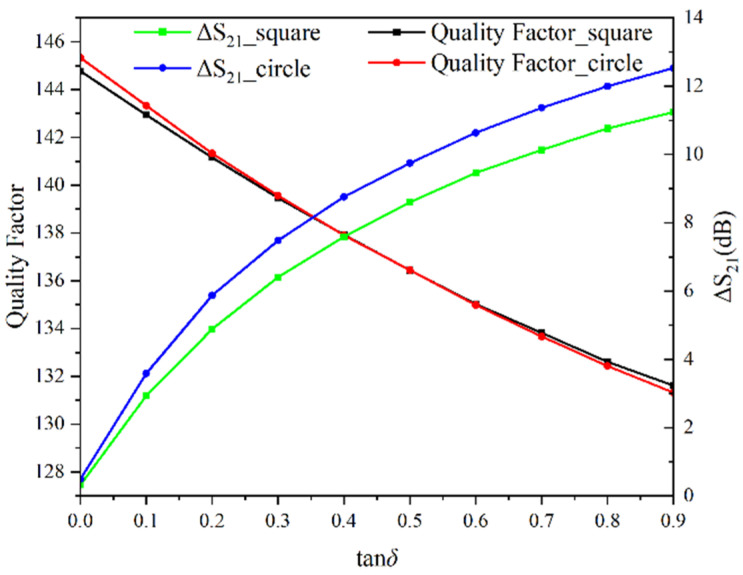
Variation of quality factor versus loss tangent for $\varepsilon_{r}^{\prime }=60$.

In Figure 6, the quality factors *Q* and $\Delta S_{21}$ of the circular CSRR structure and the rectangular CSRR structure to different loss tangent under $\varepsilon_{r}^{\prime }=60$ are plotted. It can be seen that the quality factors of the two structures are almost the same. However, the $\Delta S_{21}$ of the circular structure is 1 dB higher than the $\Delta S_{21}$ of the rectangular structure, increased by 9.7%. Considering Equation (4), it can be seen that the circular CSRR structure has higher measurement sensitivity when measuring the loss tangent.

It has to be mentioned that the comparison in this section is only for the specific case between Ref. [29] and this work, where the diameter of the circular sensor equals the side length of the square sensor. More general sensitivity needs further investigation in terms of resonance, the geometrical structure of the sensor, and even the volume ratio of the sample exposed to the sensor.

## 4. Simulation Analysis

Before extracting the permittivity from measured data, a systematic analysis has to be conducted so as to build a mapping model. This section describes simulation work using full wave simulation to conduct feasibility analysis and establish a mathematical model, so that the complex permittivity of the liquid sample under test can be expressed by the measured $S_{21}$ (resonant frequency and quality factor). Since the proposed CSRR sensor is designed for high-permittivity and high-loss liquid samples, $\varepsilon_{r}^{\prime }$ is set to 5–90 during the simulation, with a step size of 5, and the loss tangent is set to 0–1 with a step size of 0.1.

**Figure 7:**
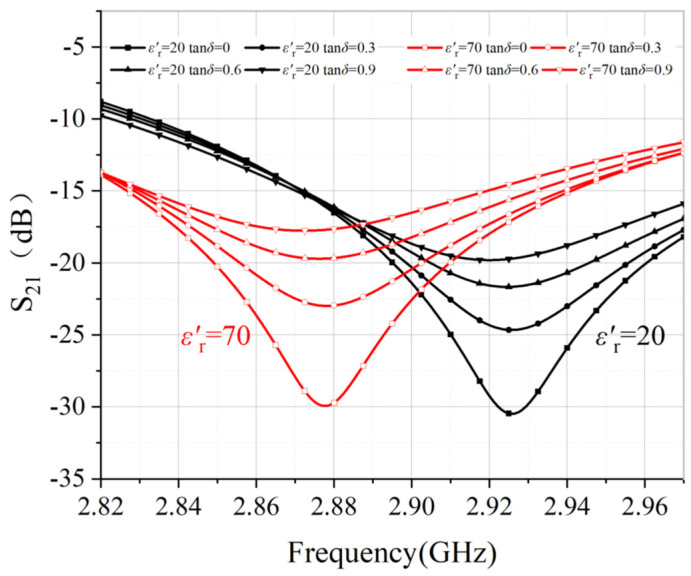
Variation of $S_{21}$ (dB) magnitude of the sensor with loss tangent value ranging from 0 to 0.9 for $\varepsilon_{r}^{\prime }=20$ and $\varepsilon_{r}^{\prime }=70$.

To ensure that the transmission coefficient $S_{21}$ is a single value function of $\varepsilon_{r}^{\prime }$ and tanδ, two groups of values $\varepsilon_{r}^{\prime }=\left(20,70\right)$ and tanδ=(0,0.3,0.6,0.9) are chosen as examples. The simulated results are plotted in Figure 7. It can be observed that: (1) higher permittivity creates lower resonant frequency, and (2) higher loss tangent reduces the resonant dips; (3) each pair of $\left(\varepsilon_{r}^{\prime },\tan\delta \right)$ only corresponds to a single curve of $S_{21}$. These facts verify that it is feasible to extract the complex permittivity from $S_{21}$.

### 4.1. Building the Frequency Function for Lossless Samples

**Figure 8:**
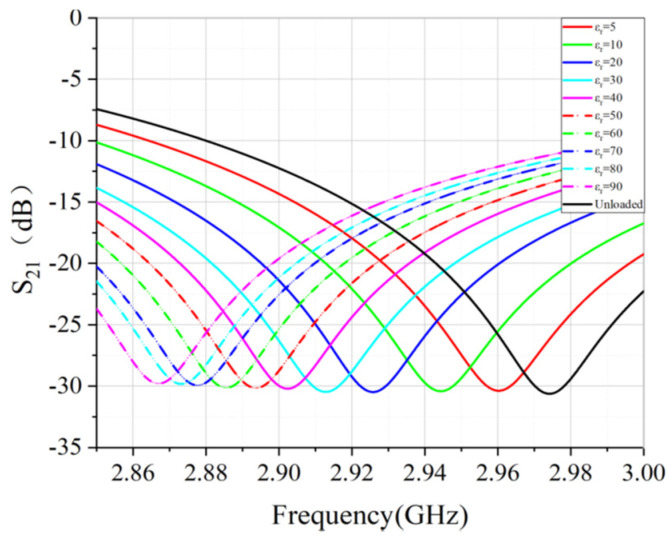
(dB) magnitude of sensor for $\varepsilon_{r}^{\prime }$ range from 5–90.

**Figure 9:**
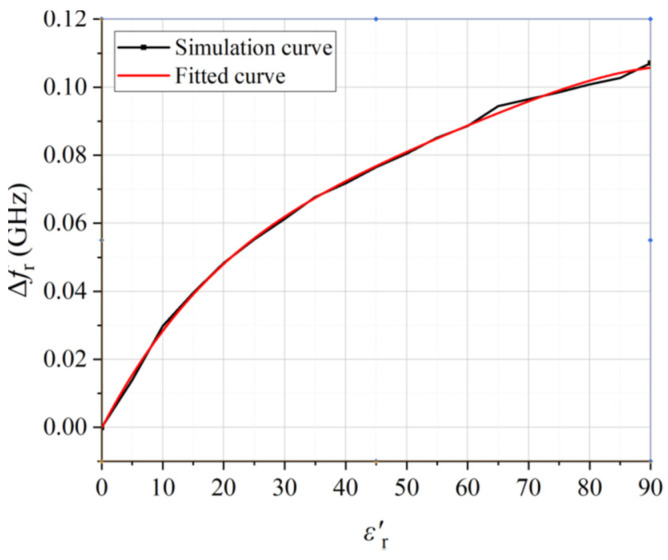
The relationship between $\varepsilon_{r}^{\prime }$ and $\Delta f_{r}$.

Since there are too many curves, only a portion of the simulated results are presented in Figure 8. For the unloaded case, the resonant frequency is 2.974 GHz. For other values of $\varepsilon_{r}^{\prime }$, the resonant frequency shifts to lower side. This reminds us that the real part $\varepsilon_{r}^{\prime }$ of the permittivity can be retrieved by observing the frequency shift $\Delta f_{r}$ relative to the unloaded condition for the lossless sample. From the simulated results, the frequency shift $\Delta f_{r}$ versus $\varepsilon_{r}^{\prime }$ is plotted in Figure 9, under the condition of the lossless case (tanδ=0). The fitted quartic equation is

(5) $$\begin{array}{l} \Delta f_{r}=f_{0}-f_{r}=-2.00691\times 10^{-4}+0.00342\varepsilon_{r}^{\prime }-6.27316\times 10^{-5}\varepsilon_{r}^{\prime }{}^{2}\\ \;\;\;\;\;\;\;+6.83277\times 10^{-7}\varepsilon_{r}^{\prime }{}^{3}-2.91958\times 10^{-9}\varepsilon_{r}^{\prime }{}^{4} \end{array}$$

Here, the coefficient of determination $R^{2}$ is 0.9998, showing a sufficiently good fit for data retrieval.

### 4.2. Building the Retrieval Function for Lossy Samples

**Figure 10:**
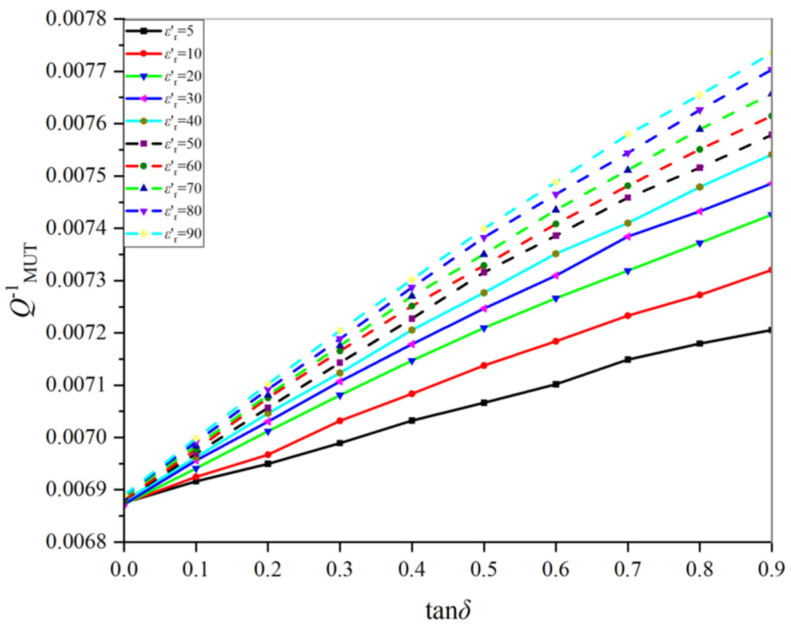
The relationship between $Q_{\mathrm{MUT}}^{-1}$ and tanδ under different $\varepsilon_{r}^{\prime }$.

In contrast, the permittivity of lossy samples involves real part and imaginary part (or loss tangent). To retrieve their permittivity, one needs to take into account both the quality factor and the corresponding resonance frequency. It can be seen from Figure 7 and Figure 8 that the resonant frequency is almost determined by the real part $\varepsilon_{r}^{\prime }$. In light of this, only $Q_{\mathrm{MUT}}^{-1}$ versus tanδ is shown in Figure 10. Clearly, higher loss tangent causes smaller $Q_{\mathrm{MUT}}$. The linearity for all cases is pretty good. Such an observation will make the retrieval results sufficiently accurate.

**Figure 11:**
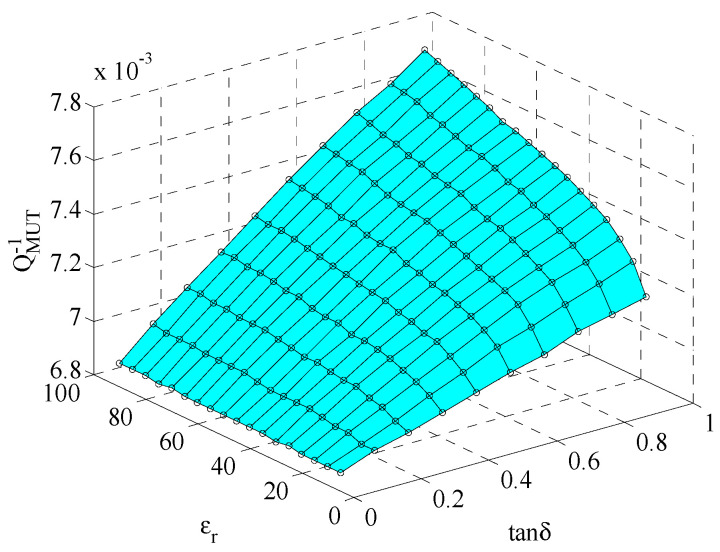
Nonlinear surface fitting for loss tangent and quality factor.

To reach a two-variable function, a three-dimensional plot is shown in Figure 11. The fitted quartic equation is:

(6) $$\varepsilon_{r}^{\prime \prime }=\varepsilon_{r}^{\prime }\frac{F(Q_{\mathrm{MUT}}^{-1},\varepsilon_{r}^{\prime })}{G(Q_{\mathrm{MUT}}^{-1},\varepsilon_{r}^{\prime })}$$

where

(7) $$\left\{ \begin{array}{l} F(Q_{\mathrm{MUT}}^{-1},\varepsilon_{r}^{\prime })=-2.75\times 10^{4}-3.79\times 10^{2}\varepsilon_{r}^{\prime }+7.50\times 10^{6}Q_{\mathrm{MUT}}^{-1}\\ \;\;\;\;\;\;-5.09Q_{\mathrm{MUT}}^{-2}+5.50\times 10^{4}\varepsilon_{r}^{\prime }Q_{\mathrm{MUT}}^{-1}\\ G(Q_{\mathrm{MUT}}^{-1},\varepsilon_{r}^{\prime })=1+1.84\times 10^{2}\varepsilon_{r}^{\prime }+2.189\times 10^{5}Q_{\mathrm{MUT}}^{-1}+0.26{\left(\varepsilon_{r}^{\prime }\right)}^{2}\\ \;\;\;\;\;\;-3.00Q_{\mathrm{MUT}}^{-2}-1.98\times 10^{4}\varepsilon_{r}^{\prime }Q_{\mathrm{MUT}}^{-1} \end{array}\right.$$

The RationalTaylor model [37] was selected to fit all points, so that the nonlinear surface covers all points in the coordinates as much as possible. In this case, the coefficient of determination *R*^2^ is 0.99943. This is also good enough for data retrieval. By using Equations (6) and (7), the complex permittivity of the sample can be readily restored.

## 5. Measurements and Results

**Figure 12:**
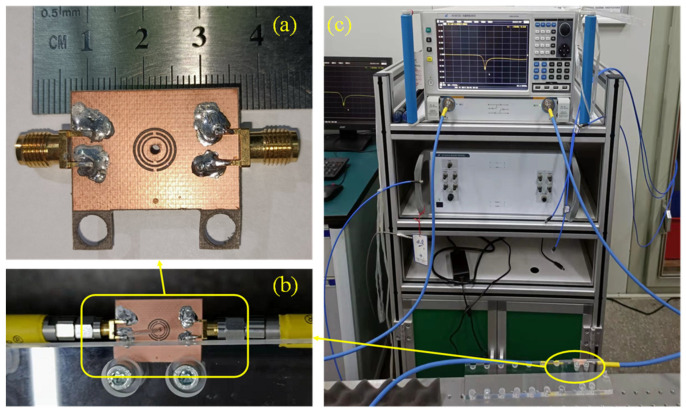
Experimental verification. (**a**) Top view of the CSRR; (**b**) The sensor is fixed on a PTFE plate; (**c**) Experimental set up.

Figure 12a,b shows photos of the sensor, which was fabricated using planar PCB technology. The sensor was made from RO3035 substrate with a thickness of 0.75 mm and a dielectric constant of 3.5 and loss tangent of 0.0015. The middle glass tube is made of fused quartz having a dielectric constant of 3.78. The tube has a diameter of 1.5 mm, a length of 75 mm, and an inner diameter of 0.3 mm. The bottom of the glass tube is sealed, and the upper end is open, which can be used to inject liquid samples into the tube. A pair of 50 Ω SMA connectors are soldered onto both ends of the sensor.

**Figure 13:**
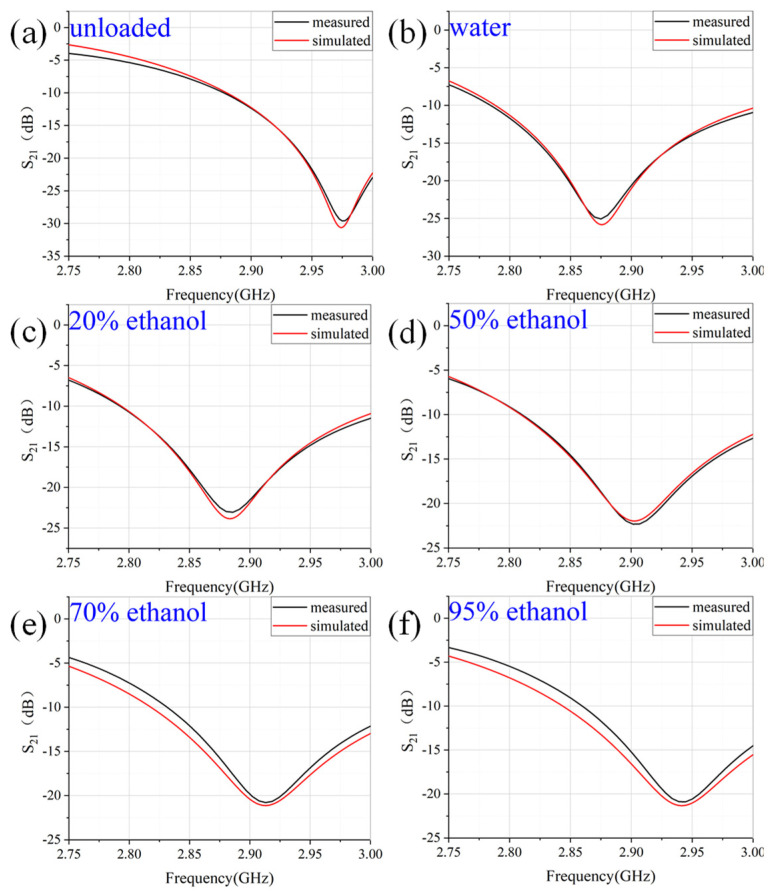
Comparison between measurement and simulation of *S*_21_ (**a**) unloaded; (**b**) 0% ethanol (water); (**c**) 20% ethanol; (**d**) 50% ethanol; (**e**) 70% ethanol; (**f**) 95% ethanol.

Measurement was conducted using a vector network analyzer (VNA) *Ceyear* AV3672D. The VNA was calibrated using a standard short-load-open-thru (SLOT) method with a 3.5 mm calibration kit. The measurement frequency range was from 2.75 GHz to 3 GHz. After calibration, the $S_{21}$ of unloaded situation was first measured. Then, the liquid samples were injected into the glass tube through syringes. In this study, the liquid sample was diluted ethanol solution with a mass concentration of 20%, 50%, 70% and 95%. These samples were injected into different glass tubes to avoid contamination. Placing the quartz glass tube filled with the measured sample to the CSRR center through the hole of the sensor and recording the $S_{21}$ for each sample. The measured data are plotted in Figure 13. From the measured data, the resonant frequency and the quality factor can be deduced. Therefore, the dielectric constant and loss tangent can be calculated by using the fitting Equations (5)–(7).

**Figure 14:**
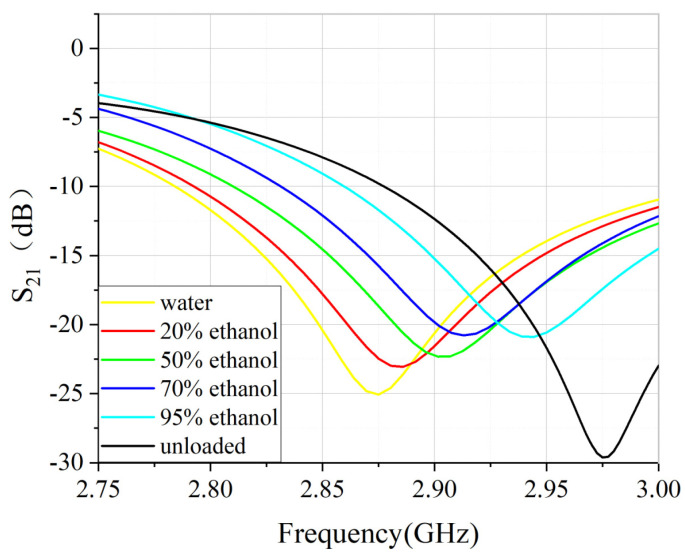
$S_{21}$ of different concentration of ethanol.

The measured data are plotted to Figure 14 to better show the tendency of $S_{21}$ with the concentration. With the increase in ethanol concentration, the resonant frequency shifts upwards, and the loss shows a decreasing tendency, which is consistent with simulations.

The dielectric constant change due to the concentration of the liquid sample can be explained using mixture theory. The mixture of water and methanol shows concentration dependent property. Water has larger permittivity and methanol has smaller permittivity. Therefore, with the increase in methanol concentration, the effective permittivity will decrease. Considering that the resonant frequency is inversely proportional to the square of the permittivity, it is seen that the resonant frequency moves towards a lower frequency side with the increase in concentration.

The values are substituted into Equations (5)–(7) and the one that obtained the complex permittivity of the sample, as shown in Table 3. The measured results have a good agreement with values reported in Ref. [38]. The calculated accuracy in comparison to the cited values is as good as 2.8%, in contrast to the values in Ref. [38]. It is noted that the worst case is the imaginary part for pure water, as seen in Table 3. This figure (2.8%) is used to represent the accuracy.

Table 4 shows the performance comparison between the proposed sensor of this work and other similar microwave dielectric sensors reported in the literature. For Refs. [24,25], the main drawback is that only the real part can be measured. The accuracy of the sensor in Ref. [25] is 11%, which is even worse than a non-resonance method. For Refs. [26,27,28], samples are in direct contact with the sensor, making them unable to measure corrosive samples. It is observed that the resonance dip becomes worse when the loss tangent increases. In comparison, this design uses a partial-filled glass tube, which is helpful to increase the resonance dip for high loss media. Compared to Ref. [38], this work shows higher relative sensitivity and provides 2.8% measurement accuracy.

From the comparison, it is seen that the proposed design is suitable for measurement of high-permittivity and high loss liquid samples. However, it has to be mentioned that for low-loss liquid samples, the accuracy is yet to be verified. In addition, since the sample is contained in a glass tube, samples are not in direct contact with sensors, which will decrease the sensitivity for low-loss samples. However, for high-loss samples, this is a good method. With this connection, there are many aspects for further study, such as the development of a sensor for the measurement of both high-loss and low-loss samples. Other topics may include the design of a sensor for very small volume samples.

## 6. Conclusions

A multi-element split-ring resonator has been designed, fabricated and verified using diluted ethanol samples for the dielectric measurement of liquids. This method used three circular ring resonators to enhance the local field so that more field interaction with samples can take place. A low-loss fused quartz was used to hold liquid so that high-loss liquid only occupies 36% volume, which enables the measurement of high-loss samples. By combining these two measures, it was demonstrated that water and diluted samples can be measured with an accuracy of 2.8%. In addition, an analytical function has been developed, facilitating the direct recovery of permittivity from measured *S*_21_. This method is also useful for the measurement of biological and corrosion samples. Further development may include systematic study on influential factors on sensitivity, and the development of sensors for the measurement of samples covering a wider range of loss and dielectric constants.

## Figures and Tables

**Table 1:** The Main Parameters for two types of CSRRs shown in Figure 1.

Parameters	a	b	c	d	e	f	g
Values (mm)	0.45	0.3	0.4	0.3	0.3	7.3	0.5

**Table 2:** The Lumped Parameters for the Equivalent Circuits.

Parameters	Rectangular CSRR	Circular CSRR
*L*	5.512 nH	5.048 nH
*C*_c_	1.563 pF	1.210 pF
*C*_r_	2.231 pF	2.187 pF
*L*_r_	1.141 nH	0.838 nH

**Table 3:** Comparison between measured results of this work and data in the literature [38].

Sample	Ref. [38]		This Work		Accuracy	
	$\varepsilon_{r}^{\prime }$	$\varepsilon_{r}^{\prime \prime }$	$\varepsilon_{r}^{\prime }$	$\varepsilon_{r}^{\prime \prime }$	$\varepsilon_{r}^{\prime }$	$\varepsilon_{r}^{\prime \prime }$
Water	79.0	10.9	80.0	11.2	1.3%	2.8%
20% ethanol	64.0	17.3	63.8	17.5	0.3%	1.2%
50% ethanol	43.0	18.0	43.1	17.9	0.2%	0.6%
70% ethanol	28.5	17.1	28.6	17.2	0.7%	1.7%
95% ethanol	11.0	9.0	10.9	9.1	0.9%	1.1%

**Table 4:** Comparison of various liquid microwave microfluidic sensors.

Referfence	Frequency (GHz)	*RS*	Accuracy	Complex Permittivity	Corrosive Sample
[24]	2.620–3.816	0.348	/	no	yes
[25]	0.86–0.91	0.069	11.0%	no	no
[26]	3.180	0.488	/	yes	no
[27]	2.189	0.98	/	yes	no
[28]	1.200–2.335	0.623–0.879	4%	yes	no
[29]	2.30–2.35	0.024	/	yes	yes
This work	2.850–2.960	0.041	2.8%	yes	yes

## Data Availability

Not applicable.
